# Clinical profile and serum Vitamin D level among dementia patients: An Insight from Ethiopia

**DOI:** 10.1002/alz.091360

**Published:** 2025-01-03

**Authors:** Yared Z Zewde

**Affiliations:** ^1^ Addis Ababa University, Addis Ababa, CA, Ethiopia; Global Brain Health Institute, University of California, San Francisco, CA USA

## Abstract

**Background:**

Vitamin D deficiency is linked with the risk of developing dementia and Alzheimer disease. There is paucity of data on serum vitamin D level among patients from tropical countries such as Ethiopia. The objective of this study is to determine the prevalence of vitamin D deficiency and associated factors among Alzheimer disease and related dementia patients in Ethiopia.

**Method:**

An institution‐based cross‐sectional study was conducted among patients presented with cognitive complaints and diagnosed with Alzheimer’s disease or Alzheimer’s disease related dementia at Lancet General Hospital in Addis Ababa, Ethiopia from November 1 – August 30, 2023. Sociodemographic and clinical data were obtained at presentation with serum vitamin D level determined subsequently. Cognitive test was assessed using the Montreal Cognitive Assessment‐Basic (MOCA‐B). Descriptive and inferential statistical analysis were done and measures of estimated crude and adjusted odds ratio with95% CI were constructed and a p value <0.05 was considered statistically significant.

**Result:**

A total of 60 adult patients with dementia were enrolled. The mean (SD) age of dementia patients was 69.4 (1.56) years and 56% were male. The prevalence of vitamin D deficiency was 70% and the mean serum vitamin D level was 23.94 (1.55) ng/ml. Amnestic variant AD was detected in 45% of the participants followed by vascular dementia (35%) and Parkinson disease dementia (10%). HIV associated dementia was diagnosed in 6% of the participants. Hypertension was the most prevalent (45%) comorbidity reported in our dementia cohort. Severe vitamin D deficiency (serum vitamin D level < 10 ng/ml) was negatively association with disease severity (p = 0.01) and increased age (p = 0.003).

**Conclusion:**

Vitamin D deficiency was prevalent in Ethiopian dementia patients. There were strong associations between severe vitamin D deficiency and dementia severity and increment in age.

